# Supplementary material for: RNAseq-Based Working Model for Transcriptional Regulation of Crosstalk between Simultaneous Abiotic UV-B and Biotic Stresses in Plants
Source: Genes (Basel). 2023 Jan 17;14(2):240. doi: 10.3390/genes14020240 (PMC9957429; doi:10.3390/genes14020240)
Supplement: Supplementary file 1 [file genes-14-00240-s001.zip › Table S1.pdf]

**Table S1 Primers used for gene specific expression analysis by qRT-PCR**

| Gene | AGI code | Forward primer | Reverse primer | size |
|---|---|---|---|---|
| Act2 | AT3G18780 | ACCTTGCTGGACGTGACCTTACTGAT | GTTGTCTCGTGGATTCCAGCAGCTT | 298 |
| CHS | AT5G13930 | GTTCAAGCGCATGTGCGACAAG | GCCGCTTCTTTGCCTAGCTTA | 165 |
| FRK1 | AT2G19190 | CCGTGAGCGGAATTACCATTAAC | GGCATCAACATCCTGTGGATG | 140 |
| F6H1 | AT3G13610 | TGAGGTGATTGCAAACGGAGAG | CGACGGTTTTCTTTCCATCGTG | 104 |
| CCoAOMT6 | AT1G67980 | ATGGCCGTATTACCGCGATAG | GGCCTTAAGACCATCGGAATG | 119 |
| HCT | AT5G48930 | CTCTTCGTTGTGGCTGATACTCCTTCTG | CCCCCACATTTAAAGAAAGTCACCTGC | 158 |
| CCR2 | AT1G80820 | CTTCCCGGAGTATCCACTTC | GCAAGCTCTTGACAGATTCG | 149 |
| MYB4 | AT4G38620 | GCCACGTTGTTTCAAGTGCA | TCCATTGCTCATGTCACTCC | 115 |
| MYB7 | AT2G16720 | CATAGCCTCCTAGGCAACAAG | GTTGATCCCTCTATGAGTGGC | 147 |
| MYB12 | AT2G47460 | TCAGACCTCAAGCGTGGAAAC | TGGTAGATGACCCGCGATTAG | 105 |
| MYB29 | AT5G07690 | TACCGGAGATAACCAGCAGTG | AAGGTTCCTTCGATGGAGGC | 166 |
| MYB32 | AT4G34990 | GTTCTCTTCCTAGATCCGCC | TCTCGTCGCAATAAGAGACC | 173 |
| MYB75 | AT1G56650 | CGAAAAGGTGCTTGGACTAC | CGGTTTAGCCCAGCTCTTACA | 107 |
| MYB111 | AT5G49330 | GGCAACAGATGGTCACTTATTG | GCTCCAGAAGACGATGAACAAG | 182 |
| bHLH78 | AT5G48560 | AACGTGGACGCTCTTGTGTC | GGGTAAGTGTTGGTAAGTGAGTG | 241 |
| bHLH UNE10 | AT4G00050 | CCAACATGATGCCAAACCCA | GCAGACATAGGATCGGGGATG | 106 |
| bZIP61 | AT3G58120 | TCAAAGACGCTCATCAAGAAGC | GTTCCTTCTCAACGGACGGT | 141 |
| WD40 | AT5G53500 | GCCAGAGGAAAACCTACCC | TCACAGGCAACCCGTAGTTC | 179 |
| WD40 | AT1G64610 | CGAGGGATTTCTCTCGCACA | AGGCCACGTTGTTGATCCTT | 151 |
| NAC003 | AT1G02220 | GTCAGTTTGGTGATGTGCTG | CCATGTTTCCCATTCCTCAATC | 135 |
| WRKY27 | AT5G52830 | CCCGTTAACCCGGTTCCTAA | TCATCTCGATCTCTCACCGCT | 263 |
| Bak1 | AT4G33430 | GAGTCAGAAAGTAGTGTCGCC | CAGGACAGCAGTCAAAGACC | 199 |
| MPK3 | AT3G45640 | CCAAGAAGCCATAGCACTCA | AGCCATTCGGATGGTTATTG | 91 |
| MPK4 | AT4G01370 | CGTTGTGCCACCCATATTT | AAAATTGAACGGCCTCACAC | 74 |
| PDF1.2 | AT5G44420 | CGCTGCTCTTGTTCTCTTTGC | TCCATGTTTGGCTCCTTCAA | 154 |
