# Supplementary figures and images for: RNAseq-Based Working Model for Transcriptional Regulation of Crosstalk between Simultaneous Abiotic UV-B and Biotic Stresses in Plants
Source: Genes (Basel). 2023 Jan 17;14(2):240. doi: 10.3390/genes14020240 (PMC9957429; doi:10.3390/genes14020240)

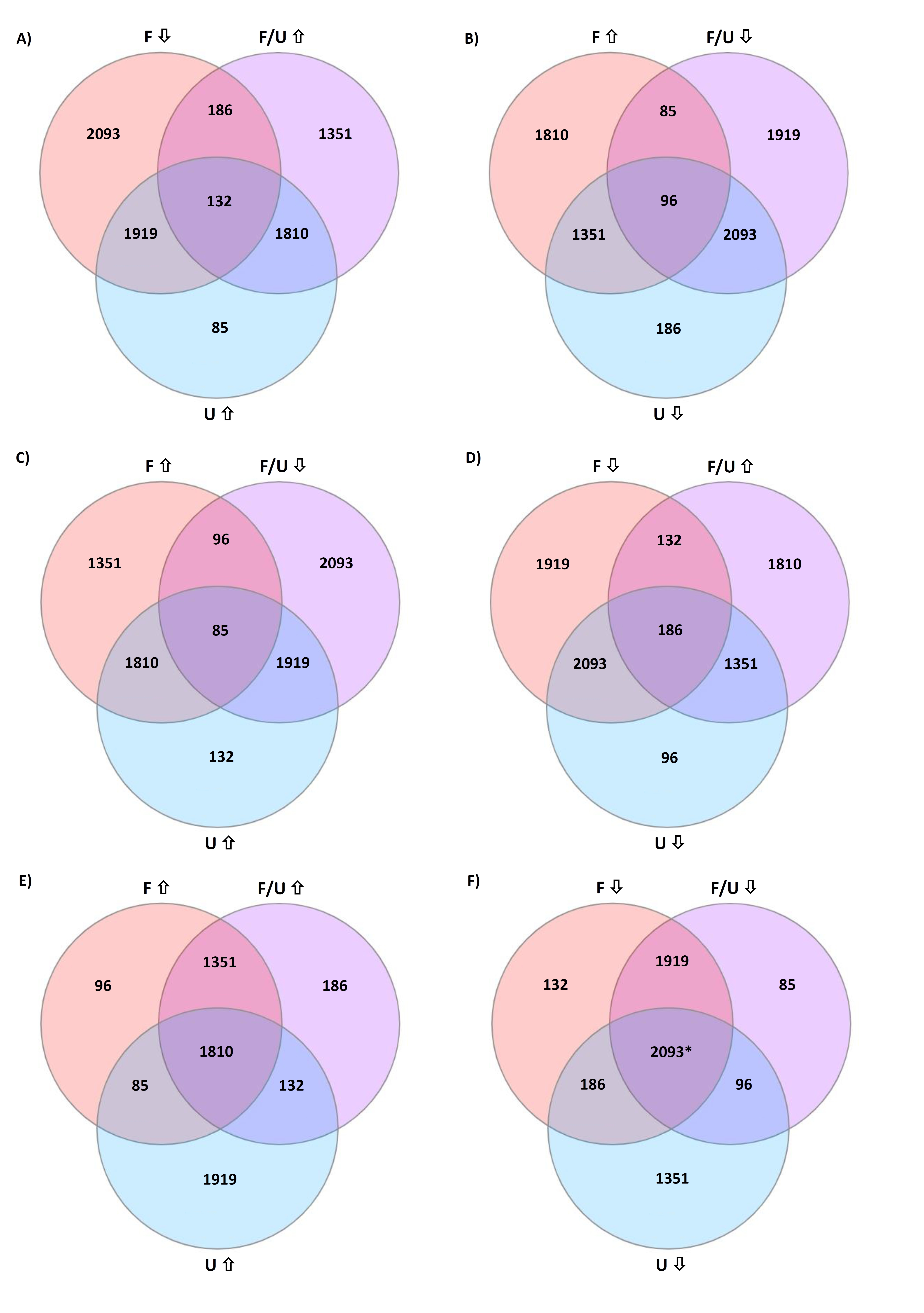

Supplement: Supplementary file 1 [file genes-14-00240-s001.zip › Figure S1.jpg]

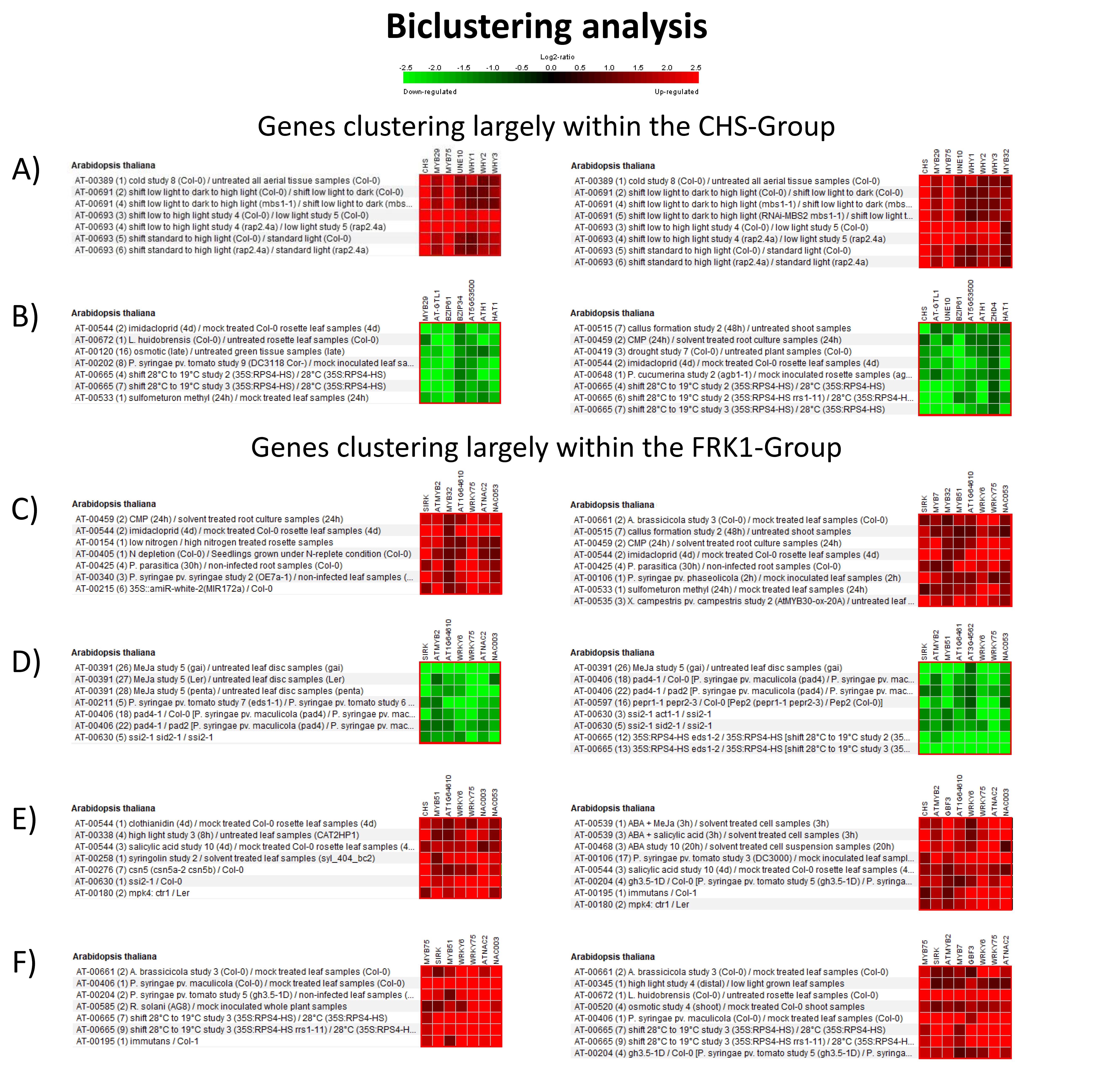

Supplement: Supplementary file 1 [file genes-14-00240-s001.zip › Figure S3.jpg]

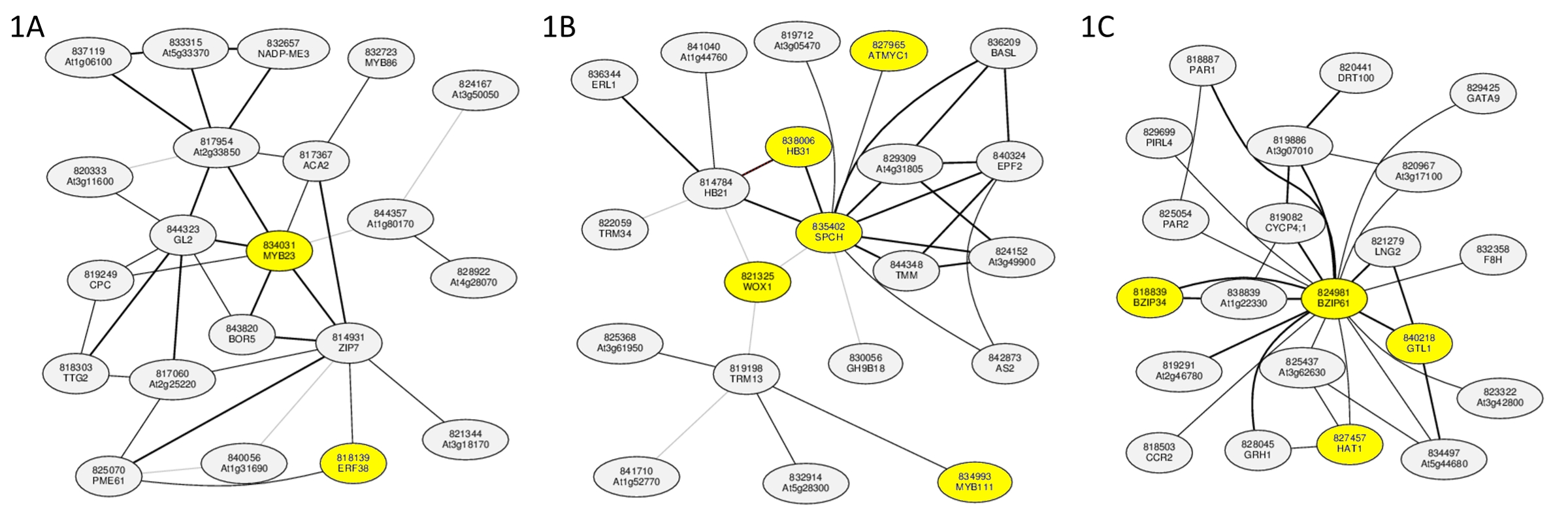

Supplement: Supplementary file 1 [file genes-14-00240-s001.zip › Figure S4.jpg]

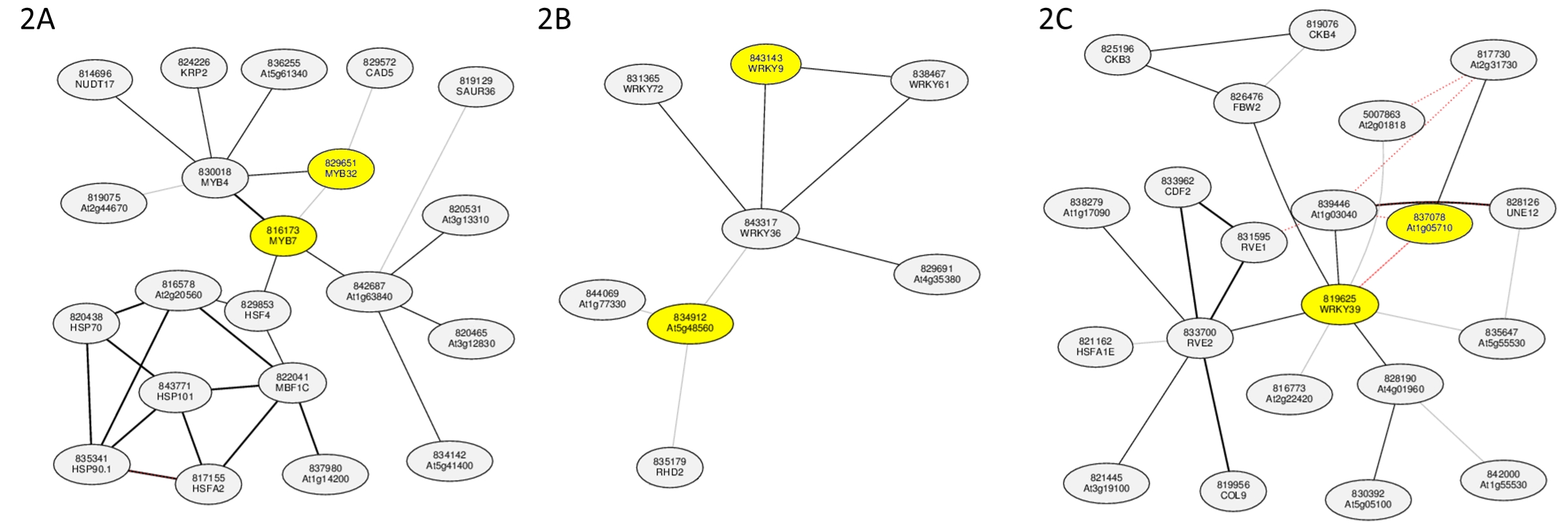

Supplement: Supplementary file 1 [file genes-14-00240-s001.zip › Figure S5.jpg]
