# Supplementary material for: RNAseq-Based Working Model for Transcriptional Regulation of Crosstalk between Simultaneous Abiotic UV-B and Biotic Stresses in Plants
Source: Genes (Basel). 2023 Jan 17;14(2):240. doi: 10.3390/genes14020240 (PMC9957429; doi:10.3390/genes14020240)

Dataset: 3240 perturbations from data selection: AT_AFFY_ATH1-0
Showing 42 measure(s) of 42 gene(s) on selection: AT-2

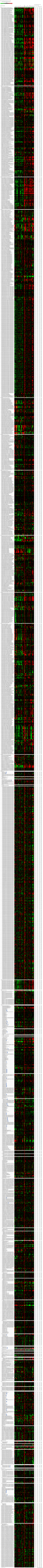

Supplement: Supplementary file 1 [file Figure S2.pdf › Figure S2.pdf]
